# Sperm Transcriptome Analysis Accurately Reveals Male Fertility Potential in Livestock

**DOI:** 10.3390/ani12212955

**Published:** 2022-10-27

**Authors:** Rhesti Indriastuti, Berlin Pandapotan Pardede, Asep Gunawan, Mokhamad Fakhrul Ulum, Raden Iis Arifiantini, Bambang Purwantara

**Affiliations:** 1 Reproductive Biology Study Program, School of Veterinary Medicine and Biomedical Sciences, IPB University, Bogor 16680, Indonesia; 2 Tuah Sakato Technology and Resource Development Center, Department of Animal Husbandry and Animal Health of West Sumatra, Payakumbuh 26229, Indonesia; 3 Department of Veterinary Clinic, Reproduction, and Pathology, School of Veterinary Medicine and Biomedical Sciences, IPB University, Bogor 16680, Indonesia; 4 Department of Animal Production and Technology, Faculty of Animal Science, IPB University, Bogor 16680, Indonesia

**Keywords:** livestock, male fertility, sperm transcriptome, transcriptomic analysis

## Abstract

**Simple Summary:**

Infertility is a problem in the livestock industry. The impact of these cases can be a long calving interval period, increasing the operational costs. Male livestock should have a breeding soundness exam before each breeding season. Even if a male passed last year, there is no guarantee he will make it this year. Therefore, the selection should be made periodically. However, male fertility potential can be predicted based on genetic characteristics, one of which is through sperm transcriptome assessment. Detection of male fertility through transcriptomic analysis provides a more sensitive and sophisticated technology assessment.

**Abstract:**

Nowadays, selection of superior male candidates in livestock as a source of frozen semen based on sperm quality at the cellular level is not considered accurate enough for predicting the potential of male fertility. Sperm transcriptome analysis approaches, such as messenger RNA levels, have been shown to correlate with fertility rates. Using this technology in livestock growth has become the principal method, which can be widely applied to predict male fertility potential in the livestock industry through the analysis of the sperm transcriptome. It provides the gene expression to validate the function of sperm in spermatogenesis, fertilization, and embryo development, as the parameters of male fertility. This review proposes a transcriptomic analysis approach as a high-throughput method to predict the fertility potential of livestock more accurately in the future.

## 1. Introduction

Prediction of male fertility is important in the livestock-breeding industry and AI centers. It is beneficial for selecting superior male candidates and re-selecting superior males for rejecting males as frozen semen producers in artificial insemination centers. In the breeding industry, in addition to female fertility and good maintenance management, male fertility greatly determines the level of cost efficiency of the production system [1]. Prediction of male fertility is based on assessing physical performance, health, reproductive organ condition, libido, mating ability, and semen quality, commonly known as a breeding soundness examination (BSE). Evaluation of semen quality generally includes parameters of sperm concentration [2], motility [3], viability [4], plasma membrane integrity [5,6], acrosome integrity [5,7], and abnormalities [5,8]. The evaluation of semen quality has begun to involve parameters for examining sperm DNA fragmentation. Researchers have reported various DNA fragmentation assay methods, ranging from chromomycin A3 staining [9,10], Hoechst, sperm Halomax kit [11,12,13,14,15], acridine orange [16], TUNEL assay [17,18,19], and SCSA [4,11,20]. This evaluation aims to investigate the competence of sperm to fertilize oocytes in the female reproductive tract. The successes of these fertilizations are compiled into data on male reproductive efficiency through the calculation of several parameters, including the conception rate, service per conception, and non-return rate. However, for superior males with a uniformly good semen quality, evaluation results still found a highly variable conception rate [21,22]. Thus, semen quality performance, according to Turri et al. [1], is not fully able to describe the male fertility level. Detection of male fertility needs more sensitive and sophisticated technological approaches to answer this challenge, one of which is through transcriptomic analysis. According to Özbek et al. [23], omics technology, especially transcriptomic analysis, extensively screen the molecular dynamics of fertility markers at both the cellular and molecular levels, with high sensitivity. Turri et al. [1] also stated that through analysis of small RNA profiles integrated with semen quality, a better understanding of sperm function was found to predict male fertility.

Transcriptomics is the study of the transcriptome—the complete set of RNA transcripts produced by the genome—using high-throughput methods to understand how the RNAs are expressed. Moreover, analysis of the RNAs transcription and expression level, function, and transcript structure from the different samples (cells or tissues) provides information about how the genes are regulated [24]. The complete set of RNA transcripts is also found in sperm. Sperm contains several RNA populations: messenger RNA (mRNA), interference RNA (RNAi), anti-sense RNA (asRNA), and micro-RNA (miRNA) [25]. Nonetheless, mature and immature sperm RNA present a series of the exon, intron, and intergenic arrays when re-mapped into the genome [26]. Sperm RNA has been identified in many mammalian species, such as humans, cattle, pigs, horses, and rodents. Some of the functions of sperm RNA include contributing during fertilization by delivering new paternal RNA to the oocyte and signaling in mitochondrial translation [27]. This sperm transcriptome also has an important role in the developmental stages of cell division in oocytes after fertilization and placenta development [26], zygote development [28], early embryonic development [29], and even epigenetic inheritance between generations [30]. Transcriptomic analysis was carried out by investigating the function of the transcriptome product (transcriptome) of sperm in the form of RNA consisting of coding RNA and non-coding RNA. Transcriptomic studies can reveal the biological function of the sperm transcriptome [14]. RNA coding is limited to messenger RNA, while non-coding RNA consists of several types, such as long non-coding RNA (lncRNA), ribosomal RNA (rRNA), transfer RNA (tRNA), t-RNA-derived small RNA (tsRNA), circular RNA (circRNA), and others. These RNAs are also transferred to the oocyte at fertilization [31].

Various methods have been used to explore the sperm transcriptome, such as gene expression analysis, RNA sequencing, and microarrays. In predicting fertility, the micro-array method has been able to answer one of the causes of infertility cases in humans since the 2000s; even in the next decade, this technology can also be applied for clinical purposes, not only for fundamental research purposes [32]. Jodar et al. [33] stated that further exploration of the sperm transcriptome could be carried out through experimental animals. However, finding specific differences between the human sperm transcriptome and other animals may be possible. This review article will discuss the use of transcriptomic analysis in predicting male fertility in livestock.

## 2. Sperm Transcriptome Analysis: Principles, Regulation, Applications, and Experimental Tools

Transcriptome analysis experiments allow researchers to characterize the transcriptional activity (coding and non-coding), focus on a subset of target genes and relevant transcripts, or profile thousands of genes at once to create a global picture of cell function. The transcriptomic analysis aims to obtain an overview of gene expression that affects certain phenotypic traits in an individual. Sperm transcriptional activity occurs during the spermiogenesis phase until the development of round spermatids [34]. The gene expression process begins with the transcription of the genetic information stored in DNA molecules, which then produces three types of RNA molecules, namely, messenger RNA (mRNA), transfer RNA (tRNA), and ribosomal RNA (rRNA). The RNA molecule that will undergo a translation into protein is mRNA. The results of this analysis are in the form of fold change values with upregulated or downregulated patterns based on references to housekeeping genes [34].

The regulation of gene expression aims to ensure that the correct proteins are made where and when needed because gene expression is essential in all living systems. The balance between the synthesis and the degradation level is a mechanism for regulating gene expression. The level of gene expression will be controlled by transcription factors (TF) when the level of synthesis is more dominant than the level of degradation. The synthesis level regulates the transcription of some genes, and other genes are regulated by on/off when the level of degradation is dominant [35]. TF is a protein that binds to specific DNA sequences and regulates gene expression. TF evolution is influenced by many factors, including epigenetic mechanisms, regulatory gene elements, and molecular cofactors [36]. Gene expression regulation is described by three layers of regulation, where the first layer is DNA that interacts with the second layer, non-coding RNA, transcription facts (TFs), and histones. Then, it becomes the third layer in the form of 3D folding results, which create the final context for gene transcription [37]. Payne et al. [38] explained that most of the regulation of gene expression is regulated by specific nucleotide sequence-binding proteins that target DNA or RNA molecules, such as transcription factors (TF) and RNA-binding proteins (RPBs). TF regulates gene expression at the transcriptional level by binding to short DNA sequences, and then activating or blocking the recruitment of RNA polymerase to the transcriptional start site.

Meanwhile, RPB regulates gene expression at the post-transcriptional stage by binding to short RNA sequences, and then regulates mRNA precursor splicing, stability, transport, translation, and decay of mature mRNA. Furthermore, they suggested that the interactions of these proteins with nucleotides could evolve into mutations at the nucleic acid-binding sites. It has the potential to alter which protein the sequence binds to, resulting in adaptive changes to the timing, level, or location of gene expression.

In detecting mRNA levels, it must be ensured that the methodology and approach used for mRNA detection are appropriate. Three primary keys must be considered in quantifying and interpreting the results of the detected mRNA expression: genes in cells/tissues that have an unequal abundance and vary from <1 to >1000. Furthermore, the intensity of the response of one gene to another varies, and gene expression can be altered for various reasons, such as by some or all specific mechanisms. There are two ways to quantify RNA expression, namely, absolute and relative. The absolute quantification method requires a standard curve. In contrast, the relative quantification method requires the difference in the fold change value between the fold change of the target gene and the reference gene (housekeeping gene). Housekeeping genes produce transcription products in constant amounts and are required for the essential maintenance of cells [39]. Some of the genes that are often used as reference genes are *ACTB* (actin or beta-actin), *TBP* (TATA-box binding protein), *cyclophilin* (PPIA, peptidyl-prolyl cis-trans isomerase A), *GAPDH* (glyceraldehyde-3-phosphate dehydrogenase), *B2M* (β-2-microglobulin), and *18S* rRNA (RNA, 18S ribosomal). *TBP*, *B2M*, and *18S* rRNA showed three reference genes with the best stability level, followed by *ACTB*, *GAPDH*, and *cyclophilin* [40].

In transcriptomic analysis, obtaining transcription products, namely, RNA, is a critical point that needs to be considered, especially from samples in the form of sperm, which have lower RNA concentrations than somatic cells [32]. It aims to obtain pure RNA from sperm without contamination from RNA somatic cells and to avoid RNA degradation [25]. Proper sperm RNA preparation is essential to get high-quality RNA. El Fekih et al. [32] stated that it could be carried out by sperm purification, sperm RNA extraction, and sperm RNA quality assessment.

To obtain a good quality and quantity of RNA, it must be ensured that the sample handling during the extraction process is carried out correctly. Kuang et al. [40] noted that RNA extraction must handle samples quickly. Suliman et al. [25] also performed the RNA extraction process by storing frozen semen on ice for 5–10 min before thawing. TRIzol solution or other Tri reagents are recommended to lyse the sample, then 2-propanol for RNA precipitation, and then ethanol. Then, to determine the quality of the extracted RNA, checking the density of the RNA using a gel or automatic electrophoresis system is more recommended than using the A260/A230 ratio. While working with RNA samples, gloves are recommended, ensuring that the entire work area is conditioned to be RNAse free, including pipettes, tip barriers, and tubes. In the complementary DNA (cDNA) synthesis stage, it is crucial to use the same reverse transcription protocol for all samples, including the amount of RNA used, the type of reverse transcriptase enzyme, and the type of primer used as the reaction volume and PCR temperature. In determining the qPCR primers, Kuang et al. [40] also suggested that the primers specific to the target gene and only to the amplified cDNA must be tested first as part of the qPCR validation process.

Extracting RNA from sperm samples is more critical than other tissues or cells. For semen samples, washing using a PBS solution is recommended; wash quickly and in cold temperatures (2–5 °C). The use of tris(2-carboxyethyl) phosphine hydrochloride (TCEP) solution with TRIzol is recommended to lyse cells more quickly during the sperm RNA-extraction process, compared to guanidium thiocyanate-phenol-chloroform (AGPC) [41]. Many factors determine the results of the transcriptomic analysis. Pang et al. [27] found that the average RNA concentration of sperm with DNase was 56% lower in the 15 min of incubation treatment with DNase (44.28 ± 8.12 ng/µL) than without DNase (101.7 ± 13.5 ng/µL) but produced a RNA quality that was not different between treatment with DNase (1.79 ± 0.03) and without DNase (1.80 ± 0.01). Using an RNA stabilizer solution is also considered significant [42]. Research on using RNAlater as a stabilizer during the isolation process has been carried out on whole sperm, mature sperm, and swim-up samples [43]. According to Parthipan et al. [44], to obtain the effectiveness of RNA isolation using high-quality RNA, it is necessary to require the amount of sperm concentration to be inputted, a suitable RNA isolation method, and a reliable method of RNA quantification and RNA quality checking. Parthipan et al. [44] reported an optimal sperm concentration of 30–40 million sperm by combining the RNA isolation methods, both RNeasy + TRIzol and PureLink + TRIzol. The combination of these methods is considered better than conventional ones (TRIzol, double TRIzol, and RNAzol RT). Research using a transcriptomic analysis approach on sperm requires several modifications to obtain the expected results. It is due to the biological complexity of sperm, including the lower sperm RNA content compared to other cells. The strong disulfide bonds in sperm chromatin make the chromatin structure more resistant to lysis during the RNA isolation process. In addition, most of the sperm RNA, except for the nucleus, is fragmented, so it is essential to isolate intact sperm RNA [27].

The transcriptomic analysis aims to measure the quantification of mRNA that can be carried out by several methods, such as quantitative polymerase chain reaction (qPCR), RNA sequencing, and microarray. The real-time PCR (quantitative PCR, qPCR) method qPCR was first reported in the 1990s [45], which was developed from the basic PCR method ten years earlier [46]. Specifically, qPCR is applied through reverse transcriptase qPCR (reverse transcriptase-qPCR, RT-qPCR), which can generate images of target gene expression from various taxa [47] by measuring the target through the emission of fluorescent waves by nonspecific fluorochromes (such as SYBR Green) or a specific hybridization probe (such as the TaqMan probe). Gene expression analysis using the qPCR method has advantages, including a relatively low cost, relatively general quantification and analysis methods, relatively easy analytical equipment to find, and the relatively small amplicon size (typically 150 bp), making it possible to use limited genomic data information. However, this method can only simultaneously analyze the expression of a limited number of genes. The selection of housekeeping genes less susceptible to bias causes the results to be biased [48].

The RNA sequencing method was used to measure the gene expression profiles and find single nucleotide polymorphisms (SNPs) and microsatellites. This RNA quantification method is performed by sequencing cDNA fragments (reverse transcription of RNA isolates) and then aligning them with newly produced transcriptomic or de novo transcriptomic references [49]. The advantages of this method are that RNA-Seq does not require genomic sequences for analysis, such as qPCR, bDNA, or microarray assays, and several studies involving de novo transcriptomic assembly are available [50,51]. Moreover, RNA-Seq is not based on hybridization, such as qPCR, multiplexed bDNA assays, or microarrays. RNA seq is also more sensitive to detect very high and very low gene abundances. However, RNA seq requires the presence of a significant cDNA library, possible bias through RNA fragmentation methods, and repeated identical reads [49]. In addition, it requires the allocation of large data storage (up to hundreds of gigabytes), and the computational process is complex.

Microarray is an analog test, hybridization-based, and a comprehensive assay that can measure a full range of transcripts in a given sample (such as a transcriptome). Microarrays typically consist of arrays of DNA or oligonucleotide probes bonded to glass or silicon slides (in a ‘‘spot’’ formation) that complement the cDNA (produced after reverse transcription and mRNA amplification in the original sample) that has been fluorescence labeled. After hybridization, the slides are scanned, and the target and background signal intensities are compared. Microarrays can be applied to determine the gene expression profiles and to detect slice variations and single-nucleotide polymorphisms (SNPs). The advantage of using microarrays for gene expression profiling is that microarrays can explore gene expression at the transcriptome level, allowing the exploration of multiple cellular pathways in parallel. However, the disadvantages of microarrays are hybridization problems, lack of primary sequence data for non-model species, data normalization, and statistical analysis. Cross-hybridization, inefficiency in hybridization, and variations in dye binding all contribute to systematic variation in microarrays [52,53]. Another drawback is that it is limited in the number of genes represented in the array, so it is not valuable enough to approach different species or tissues, such as sperm. In addition, microarrays are also less sensitive for cross-species hybridization studies [54].

The results of transcriptomic analyses are interpreted by using bioinformatics tools. Gene ontology (GO) was used to identify changes in the biological function of genes that are expressed differently between two groups with different phenotypic characteristics, including biological processes (BP), molecular functions (MF), and cellular components [55]. Differential Expression Gene (DEG) is generally associated with biological functions related to cellular processes, including the organization, function, regulation, development, growth, and proliferation of cells. The DEG list was evaluated using the DAVID software. DEG was accessed using the gene ontology database and the KEGG pathway [25]. The Kyoto Encyclopedia of Genes and Genomes (KEGG) database was used to identify signaling pathways affected by DEG analysis, such as ribosomal pathways, oxidative phosphorylation, cell cycle, proteasome, spliceosome, and oocyte meiosis [51]. Statistical analysis for getting the presentation about the biological process of gene mapping from significant Piwi-interacting RNAs (piRNAs) can be carried out using PANTHER software (http://pantherdb.org) [56]. Ablondi et al. [56] used human gene mapping as a reference. They also mentioned three crucial things about piRNA’s statistical representation: since the range size of piRNAs is around 21–32 nucleotides and the micro-RNAs’ range is about 18–24 nucleotides, they chose the minimum size of the nucleotide of the piRNAs to be 26 nucleotides to include the most piRNAs and also exclude the miRNAs at the same time; they used piRNAs of human, cattle, swine, and mouse, for which those functional genomic components are evolutionary conserved across species as the reference; and in their study, they did not include analysis of messenger RNA targets of piRNAs, which have limitations in sensitivity and specificity.

## 3. Sperm Transcriptome Analysis Approaches in the Reproduction Process

In male reproduction, regulation of gene expression occurs during spermatogenesis, to encode the protein’s roles in the structure and functions of germ cells, namely, the regulations of transcription, translation, and post-translation [57]. The intrinsic genetic program influenced by extrinsic cues regulates the transcription process through signal transduction processes and is commonly present in spermatogenic cells. The storage and utilization of transcripts produced (mRNAs) for the required proteins after transcription is generated by translational regulation. Then, post-translational regulation may be responsible for spermatid condensation through signal transduction mechanisms.

Based on the central dogma, DNA is transcribed into RNA, and then translated into proteins with specific functions, structures, and regulations in various cellular processes. Spermatozoon contains 20–30 fg of fragmented RNA, with the largest fragmentation size being 75 bp [58]. Mature sperm has transcriptional production of only 28S or 18S ribosomal RNA, which is insufficient to support translation; so, initially, sperm RNA was considered only a remnant of the wasted spermatogenesis process [34]. However, more and more studies have shown that there is an indication of the translational process in sperm cells, and there is evidence that sperm RNA contributes to fertilization and embryonic development [59,60]. Sperm RNA was first detected in ferns in the 1980s, and then a few years later in mammals. In the 1990s, through the RT-PCR, RT-qPCR, and ISH approaches, the presence of sperm mRNA was validated. For the first time in the 2000s, human sperm transcriptome profiling was carried out using a microarray approach to detect male infertility. In 2004, sperm RNA was shown to be delivered to the zygote. Small non-coding RNA was first detected in human sperm by microarray and amplified several years later by sncRNA-seq. mRNA-seq studies were carried out on sperm RNA in humans and livestock such as cattle and stallions, along with improving the Next-Generation Sequencing (NGS) technique. NGS has also been used to identify biomarkers associated with male infertility in humans or sperm quality (motility) in bulls. Epigenetic studies to modify offspring have also been carried out in mice by exposing them to extreme environmental conditions, including stress and diet. Experimental results were obtained from sncRNA profiles [61].

Mammalian sperm contains RNA sequences, including messenger RNA (mRNA), ribosomal RNA (rRNA), and small RNA (sRNA), most of which represent residual transcripts produced during spermatogenesis (Table 1). RNA-Seq characterization of bovine sperm revealed the presence of degraded and full-length transcripts, suggesting that RNA can be translated after spermatogenesis and potentially contribute to capacitation and early embryogenesis [60]. RNA in sperm is spread in several locations, namely, the outer membrane, outer nucleus, inner nucleus, and midpiece. The outer membrane contains RNAs left from the inner core during the sperm maturation process, including lncRNAs, to regulate gene expression; miRNAs, for post-transcriptional regulation; piRNAs, for protection of transposable elements tRNA fragments modified by metabolites; and rRNA fragments, for the process of spermatogenesis. The outer nucleus contains lncRNAs and miRNAs that function similarly and are residual RNAs from sperm maturation in the inner core. The inner nucleus contains RNAs useful for forming chromatin structures, namely, lncRNAs, involved in the chromatin-remodeling process, and miRNAs, to target histones and promoter/transcript start sites. Then, piRNAs bind to DNA to block transposable elements, and siRNAs maintain heterochromatin DNA. The midpiece contains mitochondrial RNA, which has a function in spermatogenesis [61].

## 4. Sperm Transcriptome Analysis of Reproductive Functions in Livestock

Many transcriptomic studies have been carried out on livestock related to reproductive phenotypes (Table 2). A correlation between the semen and fertility marker genes has been found in bovine [23,26,36,54,58,60,61,69,70,71,72,73,74,75,76], horses [25,28,77], buffalo [78,79], pig [56,64,80,81,82,83,84,85,86,87], goat [88,89], ram [90,91,92,93], and poultry [31,41,94,95,96]. The presence of RNA in sperm and semen can reflect the maturity level of sperm. The RNA concentration in each sperm cell varies, e.g., 20–30 femtograms (fg) in cattle. Through observations of sperm RNA expression levels, we can identify male fertility by understanding past processes of spermatogenesis to predict successful fertilization and future embryonic development [40]. The role of sperm mRNA is differentiated based on the centriolar and nuclear mRNA populations. Centriolar mRNA plays a role in the maintenance and development of the embryo, while nuclear mRNA helps repack oocyte chromatin [97].

Card et al. [60] stated that Holstein cattle confirmed the association between the sire conception rate (SCR) value and various levels of sperm mRNA. The gene *CCDC174*, which plays a role in sperm maturation, motility, and fertility, and is associated with an effective conception rate, was expressed higher in the group of bulls with high fertility [69]. Even the breed differences between purebred and crossbred cattle have different transcriptome profiles of sperm and testicular tissue [75]. It is presumably due to the sub-fertile/infertile conditions in the offspring of crossbred cows. The offspring of *Bos taurus* males with *Bos indicus* parents tend to have sub-fertile/infertile problems [99]. The impaired fertility of crossbred cattle is evidenced by the semen rejection rate, reaching an average value of 55% [99,100]. Furthermore, Prakash et al. [63] explored differences in global gene expression between *Bos taurus* crossbred cattle with *Bos indicus* in high-fertility and low-fertility groups using high-throughput RNA sequencing. They found that 13,536 genes were identified, with 2093 unique genes in the high-fertility group and 5454 unique genes in the low-fertility group. In the low-fertility group, there were 176 upregulated (fold change > 1) genes and 209 downregulated (fold-change < 1) genes expressed. They also found that the gene expression *ZNF706*, *CRISP2*, *TNP2*, and *TNP1*, which correlated with conception, had a much greater abundance in the high-rate fertility group based on the results of RT-qPCR analysis. A recent report from Paul et al. [78] found that as many as 709 of the 51,282 transcriptions of the transcriptomic analysis using mRNA microarrays showed dysregulation in functions located in the nucleus, nucleoplasm, and cytosol, related to binding activity, transcription, translation, and metabolic processes (MAPK signaling, oxidative phosphorylation, and ribosome pathways) in cheap buffalo sperm with low fertility.

The horses’ semen quality (motility and normal morphology of sperm) was significantly different in the fertile and sub-fertile groups. Further analysis of the transcriptome profile of horse sperm obtained as many as 437 genes that significantly differed between the two groups. *OAS1, OAS2*, and *RPL5* belong to the RNA-binding group. The genes 2’,5’-oligoadenylate synthetase 1 (*OAS1*), 2’,5’-oligoadenylate synthetase 2 (*OAS2*), interleukin-13 (*IL13*), and interleukin-22 receptor and 1 (*IL22RA1*) were found to most significantly affect biological function in horses [25].

The exploration of the chicken sperm transcriptome using a microarray was first carried out by Singh et al. [31], who found that two candidate genes as markers of RNA abundance, namely, protamine (PRM) and phospholipid C zeta 1 (*PLCZ1*), were transcribed in testes and sperm. *PRM* and *PLCZ1* are parts of the sperm RNA, a remnant of the spermatogenesis process. The transcription of sperm genes shared with the testes in chickens occurred as much as 87.3% lower than in mammals; this is because the cytoplasmic content of chickens is relatively lower than that of mammals. At the same time, transcription only in sperm is higher than in mammals due to differences in species and the number of probes in the oligo-array. A total of 2115 genes were expressed differently in fresh semen and frozen chicken semen, of which 2086 genes were downregulated while 29 were upregulated in frozen semen. *CIRBP*, *RHOA*, *HSP70*, and *HSP90* genes were selected from the results of the DEG analysis based on biological functions related to antifreeze, in which these four genes were downregulated in frozen semen using microarray and qPCR methods [50].

## 5. Future Challenges and Opportunities in the Livestock-Breeding Industry

Assisted reproductive technology, namely, AI, has been widely applied to various breeds and livestock types to improve the genetic quality of the offspring produced. However, almost half of the insemination cases show pregnancy failure due to subfertility or male infertility [101]. Accurate semen quality examination is necessary to maximize the success of insemination because a cellular-based semen quality examination has not been able to identify males with superior or inferior fertility. Therefore, there is a need for more specific and accurate tests to predict male fertility, namely, through transcriptomic analysis using RNA isolates from the studied samples. The steps to quantify gene expression consist of RNA extraction, synthesis of complementary DNA (cDNA), and quantification by quantitative PCR (qPCR). RNA isolates should have good purity; thus, with minimal contamination of various impurities. cDNA is a synthetic, single-stranded DNA resulting from a copy of the mRNA strand using the PCR technique. This PCR technique aims to amplify single-stranded DNA thousands of times according to the required amount. The PCR technique requires a forward and reverse primer as a key to the targeted sequence to be amplified.

The amount of sperm RNA that can be quantified depends on the RNA isolation method. According to Jodar [102], several essential factors need to be considered when isolating sperm RNA. These factors are the low amount of RNA in the sperm and the high amount of non-sperm contaminants in the ejaculate (~100 times more than RNA). In addition, a complete lysis process is required to release RNA from the nucleus completely, and a biological fragmentation process inhibits the use of standard controls (RIN values). Therefore, adding beta-mercaptoethanol as a reducing agent is crucial to obtaining high-quality sperm RNA.

Prediction of male fertility plays an essential role in the livestock sector in AI centers and the livestock-breeding industry. Male fertility prediction using sperm samples through transcriptomic approaches, such as microarray technology, quantitative real-time PCR, and RNA-sequencing, are used for selecting superior livestock males. One of the RNAs that play an essential role in male fertility is protamine (*PRM*), the most abundant transcript found in sperm cells [63]. The protamine transcript is only found in round spermatids and stored as inactive messenger ribonucleoprotein particles before their remodeling. The translational regulation causes the repression of protamine translationally in elongated spermatids and spermatozoa. Loss of repression leads to premature protamine translation, which results in the cessation of sperm cell development [77] and a failure to produce mature sperm [103].

Moreover, it may cause genetic sperm head defects and decrease sperm concentration. This indicates the impairment of protamine expressions can cause aberrant spermatogenesis. According to Pardede et al. [70], *PRM* gene expression positively correlated with the conception rate in Limousin, Holstein, and Ongole Grade bulls. Their expression level was significantly higher in the high-fertility group.

**Figure 1:**
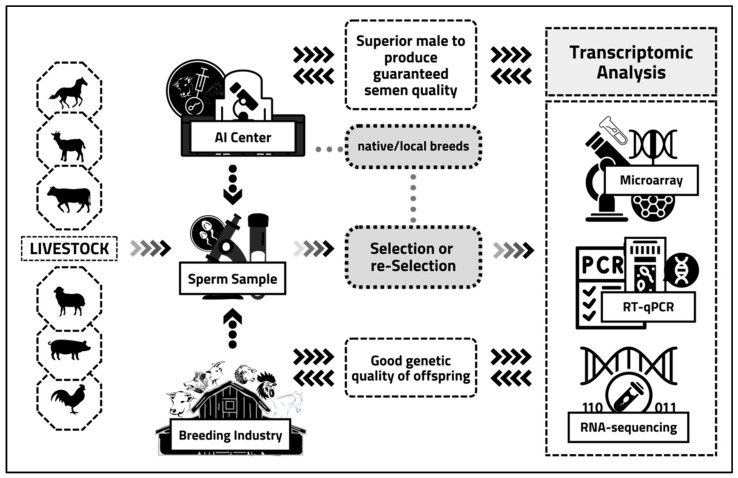
The concept of a superior male selection process is based on transcriptomic analysis in livestock. The primary sample in this new, insightful process comes from livestock sperm from the AI Center and the livestock-breeding industry. In addition, this concept also includes native/local breeds owned by several countries. Sperm samples from imported or native/local breeds are collected and used as primary specimens for the selection or re-selection process. The intended selection is for male candidates who can become superior males, while the intended re-selection is for eliminating males that were previously “considered” superior. Still, the results of insemination in the field proved to have low fertility/sub-fertility. This new selection and the re-selection process is based on transcriptomic analysis using various analytical techniques, such as microarrays, quantitative real-time PCR, and RNA sequencing. Hopefully, this transcriptomic analysis can find potential sperm transcriptomes that can be used as fertility markers to predict male fertility rates. It will undoubtedly impact the availability of superior males, whose semen quality is guaranteed and can contribute significantly to the AI Center. In addition, this new concept of male selection through a transcriptomic approach is expected to increase the genetic quality of the offspring. It will undoubtedly be very beneficial for livestock-breeding industries.

It will support AI centers to provide guaranteed high-fertility frozen semen in the future. Meanwhile, this will also improve the genetic quality of offspring produced in the livestock-breeding industry. In addition, these approaches will also help conserve and genetic improvement of native/local breeds in various animals (Figure 1).

Understanding the characteristics of the sample, standardizing the reference value of fertility marker gene expression, as well as critical points from each stage of the analysis carried out are required to predict the potential of male fertility in the livestock industry, including cattle, goat, sheep, horse, buffalo, and poultry. We also can apply these approaches for population development and genetic quality improvement of native/local breeds in the livestock industry through sperm transcriptome analysis, thereby predicting male fertility; for example, gene expression analysis of bull fertility in Indonesian native/local breeds. However, the unregistered whole-genome sequencing data from native/local breed bulls in gene banks remain a big challenge. We can use Indonesian native cattle as a sample. Based on phylogenetic analysis, cattle sequences with the closest genetic closeness to Indonesian native cattle can answer this challenge, such as the *Bos indicus* or *Bos taurus* cattle sequences. Exploration of Indonesian native cattle in the field of transcriptomic analysis have begun. Helmi et al. [104] identified the expression and characteristics of the PRM1 gene in Aceh cattle, which showed similarities (identical) to the PRM1 gene of *Bos taurus* and *Bos indicus*.

According to Fassbinder-Orth [47], there are several considerations in analyzing gene expression: technical, biological, and environmental variations. It is necessary to clearly define the purpose of the gene expression analysis to be obtained, to minimize the influence of the three variations on the analysis results. So, a good research design can be carried out by considering the differences between samples, processing techniques, and biological replication of samples.

Sahoo et al. [34] mapped the design of transcriptomic studies on cattle, goats, horses, and pigs on male fertility testing through a cDNA library analysis, RNA sequencing, or microarrays. There have been a lot of transcriptomic studies on cattle and pigs, including using epididymal semen samples, comparing testes vs. sperm, fertile vs. infertile sperm, motile vs. non-motile sperm, and fresh vs. frozen semen. The questions that need to be answered in these two livestock commodities through transcriptomic studies are related to in vitro capacitation, the incidence of apoptosis, and oxidative stress. Meanwhile, for horses and goats, only a few transcriptomic studies have been carried out, namely, coding of transcripts between sperm samples and testes in horses and coding and miRNA transcripts in goat testes. So, there are still quite a few variables that need to be answered, such as in cattle and pigs, including regarding the non-coding transcripts and sperm-specific transcripts.

Implementing sperm transcriptome analysis continuously in livestock breeding will improve the genetic quality. In the future, we may achieve a livestock industry with tremendous growth and development, reproductive efficiency and fertility, quality milk and meat production, heat tolerance, and disease resistance [105] through the combination of favorable genes with economically essential traits [106].

## 6. Conclusions

In conclusion, various types of sperm transcriptomes with potential roles in the male reproduction process have been documented. These sperm transcriptomes may be explored via multiple methods, including microarray, RNA-sequencing, and RT-PCR, as high-throughput transcriptome analysis tools. The sperm transcriptome profiles may serve as a molecular signature to identify semen with superior quality and fertility. These must validate its function in spermatogenesis, fertilization, and embryonic development. It is hoped that this will improve the conception rate of livestock by selection (and/or re-selection), crossbreeding, AI, or ATRs. This advanced analysis’s widespread and consistent implementation may provide an ideal livestock-breeding industry with economically essential traits.

## Figures and Tables

**Table 1:** The list of sperm transcriptomes and their functions in reproductive processes.

Transcriptomes	Transcript Names	Reproductive Functions	References
messenger RNA (mRNA)	*PPP1R36*	Drives autophagy during spermatogenesis	[60]
	*CRISP2*, *PEBP1*, *BSP3*, *PRKACA*, *CAPZA3*, *HSP70*	Sperm capacitation	[62,63]
	*MAP7*, *PTK2*, *PLK1S1*, *MYH9*, *PRKCZ*	Spermatogenesis and sperm function	[64]
	*DNAJC2*, *TNP1*	Sperm nuclear formation	[64]
	*CATSPER3*, *SPAG*, *COX5*, *COX1*	Sperm motility seven mitochondrial function	[64]
	*PLCB1*	Acrosomal reaction	[64]
	*PRSS37*	Sperm-egg recognition	[64]
	*HSPA1L*, *ADAM1B*, *ADAM2*, *ADAM32*	Sperm-zona pellucid binding	[64]
	*PLCZ1*	Egg activation	[64]
	*BCL2L11*, *BRCA1*	Embryogenesis	[64]
	*BSP5*, *SPADH2*	Fertilization	[64]
	*TNP1*, *RAD21*, *UBE2B*, *RAN*	Chromosomal organization	[64]
	*PAG5*, *PAG7*, *PAG10* *BCL2L11*, *MYH10*, *RBBP6*, *UBE2B*, *YBX1*	Early embryonic development	[63,64]
	*YBX1*	Embryo survival	[58]
	*TPT1*	Apoptosis, sperm functions	[63]
	*PFN1*	Oocyte maturation, fertilization, embryo development, spermatogenesis	[63]
	*ZNF706*	Sperm function	[63]
	*TSSK6*	Protein phosphorylation, sperm chromatin condensation, sperm motility, gamete function	[63]
	*TMSB10*	Sperm capacitation, fertilization	[63]
	*FABP3*	Spermatogenesis	[63]
	*IQCF1*	Sperm motility, capacitation, and acrosome reaction	[63]
	*ODF1*, *ODF2*, *SPEM1*, *MEA1*, *BCL2L11*, *PRM2*, *TNP2*	Spermatogenesis	[63]
	*RBMX*	Spermatogenesis	[65]
	*SHD*	Sperm motility	[65]
	*EFNA1*	Sperm morphology and membrane functionally	[65]
	*CDKN1B*	DNA damage, sperm apoptosis	[66]
	*ACADS*, *SCD*	Metabolic activity during cryopreservation	[66]
	*PKD2*	Sperm movement	[66]
microRNAs (miRNAs)	miR-19b, miR-let7a	Spermatogenic status	[67]
	miR-34b/c	Sperm chromatin condensation	[68]
	miR-34c	Growth, differentiation, and apoptosis of male germ cell Sperm motility	[68]
	miR-342	Sperm capacitation	[68]
	miR-19-92, miR-106b-25	Spermatogenesis, sperm quality	[68]
	Let-7a, -7d, -7e, miR-22, let-7d, let-7e	Sperm morphology and motility	[66]
	miR-29a, miR-376, miR-125b, miR-490, miR-92b-3p, miR-31a-5p, and miR-100-5p	Sperm freezing ability	[66]
	miR-3155, miR-8197, miR-6727, miR-11796, miR-14189, miR-6125, and miR-13659	Sperm fertilization	[66]
	miR-26a, miR-455-5p, miR-1	Sperm motility, apoptosis	[66]
	miR-17-92, miR-106b-25	Sperm motility, morphology	[68]
Long non-coding RNA (lncRNAs)	52lncRNAs	Sperm motility	[65]
piwi interacting RNA (piRNAs)	piR-ssc-1201188	Negative correlation with tail and neck abnormalities, proximal droplet	[56]
	piR-ssc-113649	Sperm viability, normal acrosome, and tail.	[56]
	piR-bta-16647898, piR-ssc-109407	Some parameters of sperm kinematics (sperm motility) in CASA, Sperm viability, normal acrosome, and tail.	[56]

**Table 2:** The list of sperm transcriptome analyses of reproductive functions in various animals.

Species	Samples	Phenotypes	Transcriptomic Technologies	Finding	References
Cattle	Sperm	High vs. Low Fertility	miRNA profiling miRNA sequencing	An amount of 15 miRNA was differentially expressed (9 miRNA are known, and the remaining are new)	[1]
	Sperm	High vs. Sub-fertility	Gene expression studies by Real-time PCR	*CCDC174* was confirmed as a marker for predicting bull fertility	[69]
	Sex-sorted sperm vs. unsorted sperm	High vs. Low fertility	miRNA profiling by miRNA sequencing	An amount of 85 miRNA was expressed.	[68]
	Sperm	High vs. low sperm motility	Coding and non-coding RNA sequencing	20875 protein-encoding genes were detected, and 19 were differentially expressed; meanwhile, 11,561 lncRNA were identified, and 2517 were differentially expressed	[65]
	Sperm	High vs. low fertility crossbred bulls	Global differential expression using RNA sequencing	13563 genes were found (2093 were specific in high fertile, and 5454 were specific in the low-fertility group	[63]
	Sperm	Higher vs. lower fertility	Sperm transcript profiles using RNA-sequencing	3227 transcripts (805 are unique) and 5366 transcripts (2944 are unique) were found in higher and lower fertility groups	[60]
Buffalo	Sperm	High vs. low fertility	Transcriptomic analysis using microarray technology	51282 transcripts were found, while 4050 were DE and 709 were significantly dysregulated	[78]
Sheep	Sperm	Sperm sample of Merino vs. Dohne vs. Poll Dorset	Characterize the sperm transcriptome using RNA sequencing	Dohne vs. Merino: 72 DEGs (25 upregulated and 47 downregulated)Dohne vs. Poll Dorset L 73 DEGs (41 upregulated and 31 downregulated) Merino vs. Poll Dorset: 570 DEGs (234 upregulated and 336 downregulated) These genes play an essential role in physiological functions (spermatogenesis, fertilization, conception, embryonic development, and performance of offspring production)	[91]
	Testis	Germ cells vs. somatic cells	Single-cell transcriptomic analysis of sheep spermatogenesis using single-cell sequencing (scRNA-seq)	Germ cells: several pathways of the cell cycle, gamete generation, protein processing, and mRNA surveillance were enriched Somatic cells: ribosome pathways were enriched Marker genes of germ cells: *EZH2, SOX18, SCP2, PCNA*, and *PRKD*	[92]
	Sperm	Sperm traits	Exploration of genome mutations associated with sperm traits variability using Genome-Wide Association Study (GWAS)	76 SNPs in 4 chromosomes for ejaculate concentration, 20 SNPs in 3 for ejaculate volume, 32 SNPs in 1 chromosome for ejaculate number of spermatozoa, and 23 SNPs in 17 chromosomes for spermatozoa mass motility in were found. Candidate genes for sperm traits: − Sperm motility: SLC9C1 (OAR1), TSN (OAR2), and FUT10 (OAR26) − Ejaculate concentration: *DOCK2, CPLANE1, SPEF2*, and *RAI14* (*OAR16*) − Semen volume: *SCAPER* and *PSMA4 (OAR18*) − Ejaculate number of sperm: *PARM1* and *LOC101110593* (*OAR6*)	[93]
Horse	Sperm and testis	Sperm vs. testis	Sperm transcriptome analysis using microarray analysis and RNA-sequencing	Microarray: 6761 gen/EST transcripts were found in sperm and 11,112 in testis, and 6596 of them were shared. RNA-seq: 19,257 sequence tags were mapped. 12,013 transcripts were expressed in testis	[28]
	Sperm	Fertile vs. sub-fertile	Transcriptome profiling by microarray analysis	437 DE genes were found	[25]
Pig	Sperm	Fresh vs. frozen sperm	miRNA and mRNA profiling by transcriptome and small RNA sequencing	Amounts of 567 mRNAs and 135 miRNAs were expressed differentially	[66]
	Sperm	Summer vs. winter boar ejaculates	Sperm transcriptome profiling by RNA sequencing	14 miRNAs up- and 20 miRNAs are downregulated in summer. Five miRNAs were down, and two miRNAs were upregulated in the winter	[81]
	Sperm	Embryo cleavage rate and sperm capacitation	The abundance of mRNA coding analysis using quantitative real-time PCR	*MYC, CYP19, ADAM2, PRM1*, and *PRM2* genes were more significant in the high cleavage group, while MYV mRNA was downregulated in capacitated sperm	[87]
	Sperm	Fresh vs. cryopreserved sperm (with vs. without glycerol)	miRNA expression using RT-qPCR analysis	Forty-six miRNAs were expressed. Twenty-three miRNAs were differentially expressed. Seven miRNAs were downregulated, and two were upregulated in sperm cryopreserved without glycerol. Fourteen miRNAs were upregulated, and two were downregulated in sperm cryopreserved with glycerol. Seventeen miRNAs were upregulated, but one miRNA was downregulated in cryopreserved sperm with or without glycerol.	[98]
Chicken	Sperm	Fresh vs. frozen sperm	mRNA expression using microarray analysis	2115 genes were differentially expressed, while 2086 were downregulated and 29 were upregulated in frozen sperm	[55]
	Sperm and testis	Sperm vs. testis	RT-PCR verified sperm and testis RNA analysis using microarray	Microarray: 17,524 transcripts were identified in sperm (83.7% were shared with the testis, and 12.7% were detected only in sperm). Upregulated transcripts were responsible for signal transduction (63.20%), embryo development (56.67%), and cell structure (56.25%).	[31]

## Data Availability

Not applicable.
